# Electrorheological Characteristics of Poly(diphenylamine)/magnetite Composite-Based Suspension

**DOI:** 10.3390/ma12182911

**Published:** 2019-09-09

**Authors:** Yu Zhen Dong, Hyoung Jin Choi

**Affiliations:** Department of Polymer Science and Engineering, Inha University, Incheon 22212, Korea

**Keywords:** poly(diphenylamine), magnetite, composite, electrorheological

## Abstract

Electro-responsive poly(diphenylamine)(PDPA)/Fe_3_O_4_ composite particles were prepared by the synthesis of PDPA particles using a chemical oxidative polymerization technique followed by loading nano-sized Fe_3_O_4_ particles onto PDPA via a chemical co-precipitation process. The morphological image of the PDPA/Fe_3_O_4_ particles was characterized by scanning electron microscope and transmission electron microscope. The crystalline structure was scrutinized by X-ray diffraction. The rheological characteristics of the suspension composed of PDPA/Fe_3_O_4_ particles suspended in silicone oil were investigated by a rotation rheometer, demonstrating standard electrorheological (ER) characteristics with a dramatic increase in shear stress and dynamic moduli under the application of an electrical field strength. The shear stress curves under an electrical field could be described using the Bingham model and the yield stress showed a power-law relationship with the electric field strength with an exponent of 1.5, following the conduction model. Furthermore, the frequency-dependent dielectric behaviors of the PDPA/Fe_3_O_4_ ER suspension was tested using an inductance (L)-capacitance (C)-resistance (R) (LCR) meter. The dielectric properties were well described using the Cole–Cole equation and were consistent with the results of the ER experiments.

## 1. Introduction

**Scheme 1:**
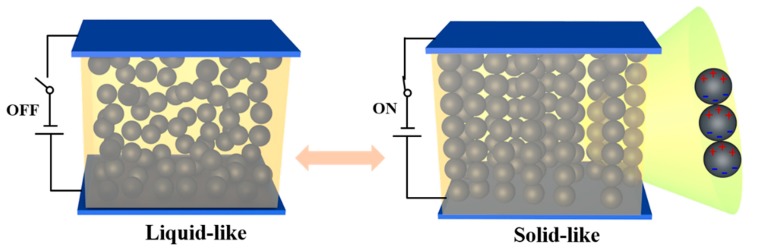
Schematic diagram of transition between liquid-like and solid-like state for electrorheological (ER) fluid.

Electrorheological (ER) fluids belong to a class of “smart” materials, which are generally suspension systems consisted of semiconducting or polarizable particles suspended in a continuous phase medium, primarily for anhydrous insulating liquids, such as silicone oils, mineral oils, etc. [1]. Their rheological properties exhibit dramatic and rapid changes from a liquid-like to a solid-like phase upon the application of external electrical fields. The mechanism for this phenomenon is still being explored, but the most accepted one is the polarization mechanism. As given in Scheme 1, while without an electrical field, the particles in the ER fluid disperse freely in the carrier liquid and assume a liquid-like state, with an input electrical field, the dipoles of the particles can be polarized instantly and arrange to make a chain-like form with the direction of the electrical field due to the induced electrostatic force among the particles [2,3,4]. Therefore, the ER suspension becomes a solid-like phase, exhibiting different rheological behaviors compared to a liquid-like ER suspension without an external electrical field. The changes in rheological properties include an increased shear viscosity, appearance of yield stress and increased dynamic modulus, where the ER fluid is similar to Bingham plastics [5,6]. In particular, chain-like structures are released as the electrical field is removed, allowing the ER suspension to recover to a liquid-like phase [7]. This tunable transition of ER fluids through the addition and removal of an external electrical field is believed to have a very large application potential in many industrial areas [8,9]. As ER fluids are developed continuously and the ER mechanism is understood more completely, in addition to being applicable to conventional components, such as dampers, brakes, switches, etc., ER fluids are gradually attracting attention in the fields of intelligent robotic systems, crude oil transportation, and food processing [10,11,12].

A range of materials, such as cellulose, starch, clay, oxide mineral, carbon-based materials (carbon nanotube, graphene oxide, etc.), conducting polymers, poly(ionic liquid) (PIL), etc. have been studied for the development of ER materials [13,14,15,16,17,18,19]. Compared to extrinsically polarizable materials that need to absorb water to activate ER effects, materials that are intrinsically polarizable or conducting are favored because of their better prospects in industry. Among them, the intrinsic conductive polymers have drawn extensive attention owing to their processability, low specific density, flexibility, etc. Most of these polymers are conjugated systems, and the π-bonded electrons in the polymer chain have low ionization potentials and high electron affinities. This causes the π-bonded electrons to easily escape from the orbit to form enough charge carriers, and the π-electron orbits overlap to form a conduction band to provide a channel for the movement of charge carriers, thereby making the polymer conducting. In particular, the conductivity of such conducting polymers can be controlled using a doping and de-doping process to suit different applications [20,21]. By taking an advantage of doping and de-doping, electrons can be added to the empty orbit of the polymer or the electrons can be pulled away from the occupied orbital. Therefore, the energy level of the original π-electron energy band is changed, resulting in a changed energy level difference between the energy bands. Consequently, the electrical conductivity can be changed. Conductivity too low usually leads to undesirable ER effects, and conductivity too high can easily cause short circuit problems, therefore a suitable conductivity is an important factor for its introduction as an ER material. Research on conducting polymers in the field of ER has also reported great achievements, such as polyaniline (PANI), polypyrrole, polyindole, poly(3,4-ethylenedioxythiophene), etc. [22,23,24,25,26]. Nonetheless, the electrical conductivity of these polymers in their pristine state is usually too high to be used directly as ER materials. Therefore, for use in ER fluids, these conducting polymers usually require an additional de-doping process.

As a type of conducting polymer, poly(diphenylamine) (PDPA) has also drawn attention owing to its electrochemistry, optical property, and higher solubility in organic solvents compared to PANI, and other conducting polymeric materials [27]. Moreover, the appropriate electrical conductivity of PDPA makes it suitable as an ER material because it does not require any de-doping process to prevent short circuits and is enough to generate sufficiently strong chain-like form with an input electrical field. Furthermore, recent research has shifted gradually to conducting polymer composites because they not only improve certain inherent limitations of polymers, but also enrich the properties of materials, such as chemical stability, mechanical properties, and thermal stability [28,29]. In particular, composite materials of conducting polymer and inorganic nanoparticles can impart special properties to the composites and enhance their application potential in many fields [30]. A wide range of this type of organic–inorganic composite material have been synthesized and adopted as excellent ER materials, including PANI/laponite [31], poly(3,4-ethylenedioxythiophene)/SiO_2_ (PEDOT/SiO_2_) [32], core–shell structured Fe_3_O_4_-polypyrrole (Fe_3_O_4_–PPy) [33], SiO_2_/PPy [34], Fe_2_O_3_/PANI [35], TiO_2_/PANI [36,37], and Fe_3_O_4_@PANI [38]. These materials exhibit excellent ER properties and yield stresses of up to several hundred Pa under an electric field. However, these materials all require the process of de-doping with either sodium hydroxide solution or aqueous ammonia solution before preparing the ER fluid. In contrast, one of the novelties of the materials prepared in this work was that it was expected to have suitable conductivity for their ER application without any de-doping process. Nano-sized Fe_3_O_4_ is often used as an inorganic component and is also used widely in chemistry, food, and medicine [39,40,41] because of its soft magnetic properties, biocompatibility, low cost, and easy preparation. In addition, it has been used as ER materials [42].

In this study, organic–inorganic PDPA/Fe_3_O_4_ composite particles were synthesized by loading nano-sized Fe_3_O_4_ nanoparticles on a conducting PDPA matrix using a simple chemical co- precipitation method and adopted in an ER suspension. The behaviors of the PDPA/Fe_3_O_4_ composite particles were characterized and then their ER properties when suspended in silicone were investigated.

## 2. Materials and Experimental Methods

### 2.1. Materials

Diphenylamine (DPA) (Sigma-Aldrich, St. Louis, MO, USA), ammonium persulfate (APS) (Daejung Chem., Siheung, Korea) and hydrochloric acid (HCl) (Junsei Chem., Tokyo, Japan) were used for the synthesis of PDPA first. In the process of loading Fe_3_O_4_ on PDPA, ferric chloride hexahydrate (FeCl_3_·6H_2_O) (Sigma-Aldrich, St. Louis, MO, USA), ferrous sulfate heptahydrate (FeSO_4_·7H_2_O) (Sigma-Aldrich, St. Louis, MO, USA), and ammonia solution (NH_4_·OH, NH_3_: 28–30% (mol/mol) (Junsei Chem., Tokyo, Japan) were used. Ethanol (Samchun Chem., Pyeongtaek, Korea) and distilled water were adopted as solvents. The continuous phase of the ER suspension was silicone oil (50 cSt, Shin-Etsu, Tokyo, Japan).

### 2.2. Synthesis of PDPA

At first, PDPA particles were fabricated through a chemical oxidative polymerization process, as proposed in the previous study [43]. DPA (6.0 g) monomer was dissolved in ethanol (150 mL) with intensive magnetic stirring for a few minutes to obtain a homogeneous solution and it was then transferred to a three-neck glass reactor (500 mL). Once an aqueous HCl solution (3 M, 120 mL) was input, the reaction mixture was maintained at 5 °C by a refrigerated bath under stirring. Concurrently, a solution of APS (3.01 g) dissolved in the aqueous HCl (3 M, 15 mL) was added dropwise to the reaction mixture. The resulting reaction mixture was stirred continuously for an additional 16 h. The product particles were separated and cleaned several times with ethanol and distilled water using a centrifuge. The precipitate was dried completely in a vacuum oven (60 °C for 12 h).

### 2.3. Synthesis of PDPA/Fe_3_O_4_

**Scheme 2:**
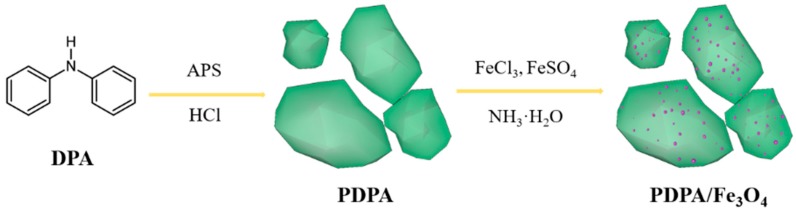
Synthetic route of poly(diphenylamine)(PDPA)/Fe_3_O_4_ composite.

The PDPA/Fe_3_O_4_ composite nanoparticles were then prepared by loading magnetite on PDPA via a chemical co-precipitation process. PDPA (1 g) was suspended in a 50% aqueous solution of ethanol and mixed with 40 mL of an aqueous solution (0.5 M FeCl_3_ and 0.25 M of FeSO_4_). After heating the mixed solution to 60 °C, 10 mL of aqueous NH_3_ solution was added, and the reaction was continued for 2 h. The precipitated product was collected by magnetic separation and then cleaned several times with ethanol and dried completely at 60 °C for 12 h. Scheme 2 presents a schematic diagram of the synthetic route of the PDPA/Fe_3_O_4_ composite.

### 2.4. Characterization

The density of the PDPA/Fe_3_O_4_ particles tested via a gas pycnometer (AccuPyc 1330, Micromeritics Instrument Corp., Norcross, GA, USA) was 2.44 g/cm^3^, while the electrical conductivity was obtained to be 5.9 × 10^−8^ S/cm via a low resistivity meter (Loresta-GP MCP-T610, Mitsubishi Chem., Tokyo, Japan) and a square probe (electrode distance: 1.5 mm) (MCP-TPQPP, Mitsubishi Chem., Tokyo, Japan). For the measurement, the PDPA powder was pressed into a disk with a diameter of 13 mm. The PDPA/Fe_3_O_4_ ER suspension was prepared by suspending PDPA/Fe_3_O_4_ (particle concentration of 10 vol%) in silicone oil.

## 3. Results and Discussion

### 3.1. Characterization of Synthesized Materials

**Figure 1:**
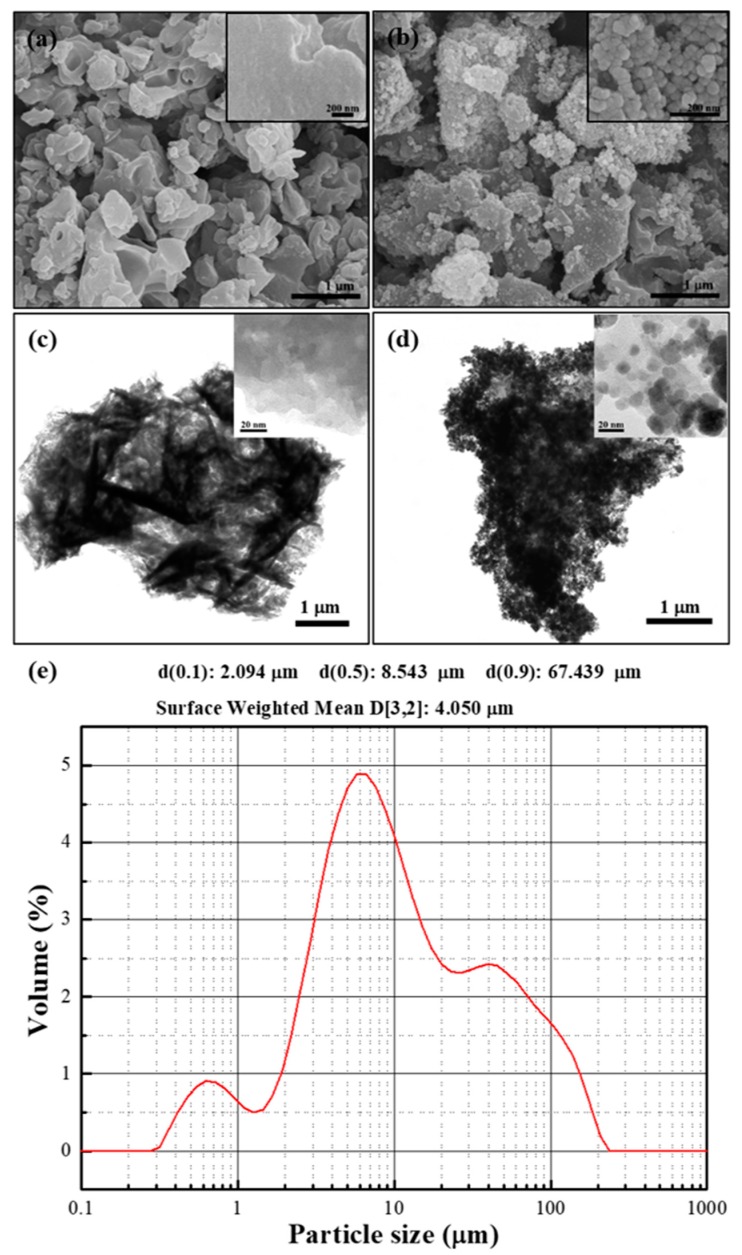
(**a**,**b**) SEM, (**c**,**d**) TEM images of PDPA and PDPA/Fe_3_O_4_, and (**e**) particle size distribution of PDPA. (The inset shows partial enlargement).

Morphological images of PDPA and PDPA/Fe_3_O_4_ were investigated via a scanning electron microscope (SEM) (SU-8010, Hitachi, Tokyo, Japan) by adhering the powder to the sample holder with a double-sided carbon tape. As shown in Figure 1a, PDPA showed irregular shapes with a size distribution ranging from approximately 0.3 microns to a few microns. Simultaneously, the particle size distribution of PDPA (Figure 1e) measured by the laser light scattering on a Malvern particle analyzer (Malvern Mastersizer 2000, Malvern Instruments, Worcestershire, UK) also showed that the smallest particle size was about 0.3 microns and the surfaced weighted mean diameter (D[3,2]) was 4.05 microns. Of the particles 50% were less than 8.54 microns, while particles over a few tens of microns are believed to be due to incompletely dispersed PDPA agglomerates, similar to the fact that large particles were not observed in the SEM image. The insets in Figure 1a,b showed partial enlargements of the surface of the PDPA and PDPA/Fe_3_O_4_ composite, respectively. The surface of PDPA/Fe_3_O_4_ was rougher than PDPA and spherical nanoparticles can be observed clearly. In order to further determine the morphology and structure of the materials, both PDPA and PDPA/Fe_3_O_4_ powders were dispersed in water by sonication, and then their dispersions were added dropwise on a copper mesh, sufficiently dried, and observed by a transmission electron microscope (TEM) (CM-220, Phillips, Amsterdam, The Netherlands). Figure 1c,d present the TEM images of the PDPA and PDPA/Fe_3_O_4_ composites, respectively, and the insets show the partial enlargements. Many spherical particles with diameters between 10 and 20 nm in enlargement of the PDPA/Fe_3_O_4_ can be seen clearly. The Fe_3_O_4_ granules on the surface of PDPA can increase the roughness and specific surface area, which is believed to contribute to an enhanced ER effect.

**Figure 2:**
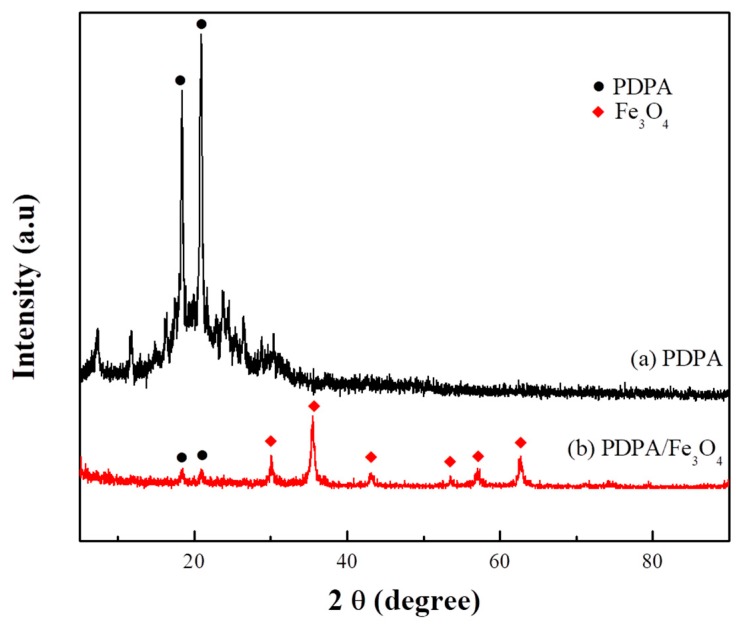
XRD spectra of (**a**) PDPA and (**b**) PDPA/Fe_3_O_4_.

Powder X-ray diffraction (XRD) (DMAX-2500, HORIBA, Kyoto, Japan) test of the PDPA and PDPA/Fe_3_O_4_ composite was performed from 5° to 90° 2θ using Cu-Kα. Figure 2 presents the XRD data of both as-synthesized PDPA and PDPA/Fe_3_O_4_ composite. Two distinct XRD peaks at approximately 18° and 21° 2θ for PDPA, and there was no XRD peak in the range of 40° to 90° 2θ. The pattern of the PDPA/Fe_3_O_4_ composite showed corresponding peaks for the (220), (311), (400), (422), (511), and (440) planes, which match with the cubic spinel crystal structure for Fe_3_O_4_ (JCPDS Card. No. 88-0315). The crystallite size (D) of Fe_3_O_4_ was determined from the following Scherrer equation [44]:

(1) $$D\;=\;\frac{k\lambda }{\beta cos\theta }$$

where *k* is a constant value of 0.89; *λ* is the radiation wavelength (1.54184 Å for Cu-Kα); *β* is the full width at half maximum (FWHM) of the XRD peak in radian, which is equal to 0.5° (π/360 radians) at the highest intense peak at (311); *θ* is the Bragg diffraction angle, and the value at the highest intense peak was 17.77 ° (17.77 π/180 radians). Therefore, the crystallite size was calculated to be 16.51 nm. XRD of the composite revealed not only the characteristic peaks of the Fe_3_O_4_, but also the peaks of PDPA at approximately 20°.

### 3.2. Electrorheological Effect

#### 3.2.1. Formation of Chain-Like Structures

**Figure 3:**
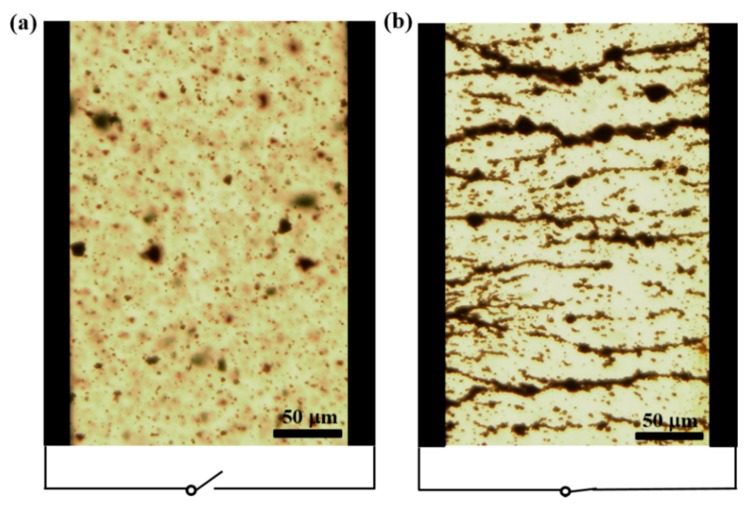
Optical microscopy (OM) images of PDPA/Fe_3_O_4_ particles suspended in silicone oil without (**a**), and with (**b**) the electric field (0.75 kV/mm).

Optical microscopy (OM) was used to observe the transition of the suspension state for PDPA/Fe_3_O_4_ nanoparticles in silicone oil under an electrical field. Figure 3a shows the PDPA/Fe_3_O_4_ nanoparticles dispersed without an electrical field, in which the nanoparticles were dispersed freely in the oil. When an electrical field was applied (Figure 3b), however, dipole changes of the particles formed, and particles arranged into chain-like structures in an electrical field direction due to electrostatic attractive forces between neighboring particles. In addition, during the direct observation with the OM, it was found that at the moment of the application of an electric field strength, the originally dispersed particles began to move rapidly to form a chain structure and reached a stable state within a few seconds, and over time, adjacent chain structures tended to gather together to form a chain with certain thickness. This may be because the direction of the dipoles was not completely coincident with the direction of the electric field, and the dipole interaction perpendicular to the direction of the electric field caused the aggregation of adjacent chains, however, this phenomenon may increase the strength of the chain structure. Furthermore, it can be also noted that even though a dynamic time-dependent evolution of the chain structure under applying the electric field is considered to be important for understanding the ER mechanism, this work has been seldom examined either via theoretically [45] or experimentally [46,47]. No direct observation has been performed.

Because of the formation of chain-like structures, the ER fluid can transform to a solid-like state from an initial fluid-like state by applying an external electrical field. Therefore, with an input electrical field, the ER suspension can exhibit obvious yield stress at a low shear rate range and shear-thinning behaviors of shear viscosity under a shear flow with a direction perpendicular to the electrical field, which can be corroborated in the following ER tests.

#### 3.2.2. Steady Shear Tests

**Figure 4:**
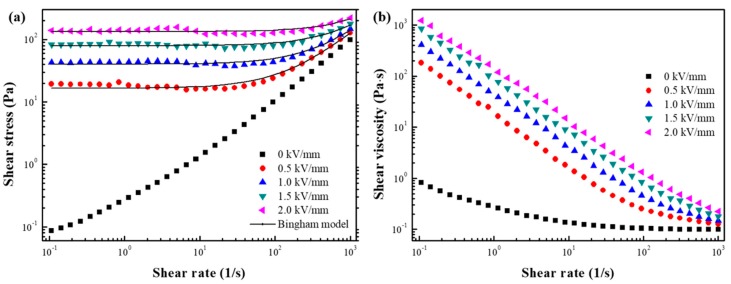
Shear rate-dependent (**a**) shear stress and (**b**) shear viscosity curves of PDPA/Fe_3_O_4_-based ER fluid (10 vol%, 50 cSt silicone oil) under different electric field strengths.

The ER characteristics for the PDPA/Fe_3_O_4_ composite ER suspension were investigated by a rotational rheometer (MCR 300, Anton Paar, Graz, Austria) with a cylindrical Couette-type geometry (CC17/E) of 0.71 mm gap distance. To investigate the ER properties in the presence of electrical field, a DC voltage generator was attached to the rheometer. Steady shear tests were measured using a controlled shear rate (CSR) mode with a shear rate ranging from 10^−1^ to 10^3^ 1/s at room temperature. Figure 4 shows both shear stress (Figure 4a) and viscosity (Figure 4b) as a function of shear rate with an input electric field (0–2.0 kV/mm). Without an electric field, the shear stress presented an approximately linear relationship with the shear rate, indicating a similar trend to that of a Newtonian fluid [48]. On the other hand, under an electrical field, the shear stress increased significantly and showed a plateau region in a low shear rate range, which is consistent with Bingham fluid characteristics [5,49]. Here, the particles were being polarized under the action of an electrical field, and an electrostatic force was generated to cause the particles to build a chain-like structure following the electrical field direction, thereby changing the ER suspension from a fluid-like to a solid-like phase. Under shear flow, when an electrical field was applied, competition between the hydrodynamic and electrostatic force in the ER suspension occurred. At the low shear rate range, the electrostatic force was predominant, and the deformation or damage to the chain structure caused by the shear flow could be recovered in time. As the shear rate increased, the electrostatic force gradually failed to recover the chain structure in time, and the particles begun to flow. The dynamic yield stress is known to be a minimum shear stress, in which the chain structure starts to be destroyed and the suspension can flow with shear flow. When the shear rate is sufficiently large, the hydrodynamic force becomes significant. The chain-like structures were almost completely broken and could not be recombined, where the ER suspension exhibited a similar property to a Newtonian fluid when the shear rate became larger than 100 (1/s), as shown in Figure 4a.

Figure 4b presents shear viscosity curves. Without an input electrical field, the sample fluid exhibited weak shear-thinning characteristics, and as the shear rate increased, it gradually approached Newtonian fluid features. Under an input electrical field, however, the shear viscosity increased by hundreds to thousands of times compared to that without an electrical field, and exhibited significant shear-thinning as the shear rate increased.

Furthermore, as presented in Figure 4a, the shear stress curves under an electrical field can be described using Bingham fluid model [50], which can be represented as

(2) $$\tau_{s}=\tau_{dy}+\eta_{\infty }\dot{\gamma },\;\tau_{s}\geq \tau_{dy}$$

(3) $$\dot{\gamma }=0,\;\tau_{s}{<\tau }_{dy}$$

where $\tau_{s}$ is the shear rate dependent shear stress; $\dot{\gamma }$ is the shear rate; $\eta_{\infty }$ is the plastic viscosity; $\tau_{dy}$ is the dynamic yield stress, which generally shows a power-law relationship with an electrical field strength. Table 1 lists the fitted parameters of the Bingham fluid equation for the PDPA/Fe_3_O_4_ ER fluid. Note that pure PDPA was also reported to be an ER material [43], and compared with a PDPA-based ER fluid, the PDPA/Fe_3_O_4_-based ER suspension showed a more stable shear stress in the low shear rate range (<10 1/s), whereas the former showed reduced shear stress as the shear rate increased. Meanwhile, the electric field limit of PDPA/Fe_3_O_4_-based ER fluid that can be applied is 2.0 kV/mm, which is lower than that of the PDPA-based ER suspension (2.5 kV/mm). This is because the electrical conductivity of PDPA/Fe_3_O_4_ (5.9 × 10^−8^ S/cm) was higher than that of PDPA (8.913 × 10^−11^ S/cm), and its particle concentration prepared at 10 vol% was larger than that of the PDPA ER fluid (5 vol%).

**Figure 5:**
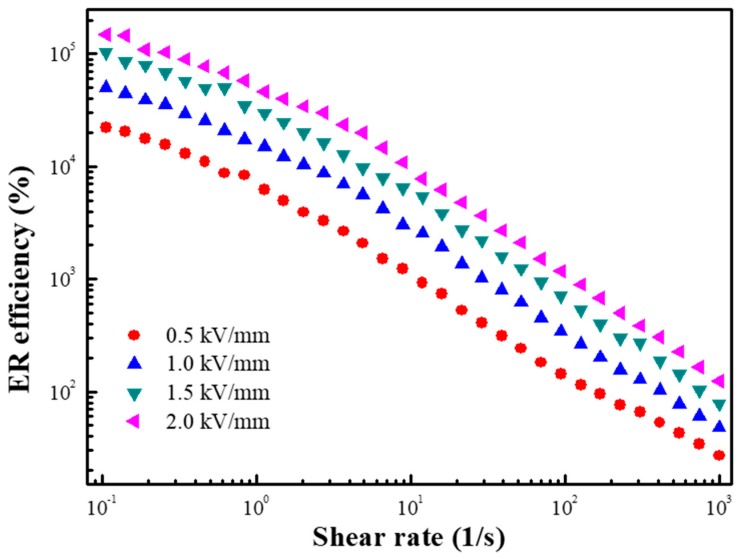
Shear rate-dependent ER efficiency of PDPA/Fe_3_O_4_-based ER fluid (10 vol%, 50 cSt silicone oil) under different electric field strengths.

Furthermore, to evaluate its ER effect, the ER efficiency (*e*) was calculated using the shear viscosity at a specific electric field using the following equation:

(4) $$e\;=\;\frac{\eta_{E}{-\eta }_{0}}{\eta_{0}}\;\times \;100\%$$

where $\eta_{E}$ and $\eta_{0}$ represent the shear viscosity at an input electrical field strength of *E* and zero-field, respectively. As given in Figure 5, the ER efficiency increased with an increased shear rate, and the value was approximately 10^6^% at an electrical field strength of 2.0 kV/mm, which indicates that the PDPA/Fe_3_O_4_ particles formed strong chain structures with an input electrical field. Meanwhile, as the shear rate increased, the chain structures were gradually destroyed and the ER efficiency was reduced continuously.

#### 3.2.3. Dynamic Oscillation Test

**Figure 6:**
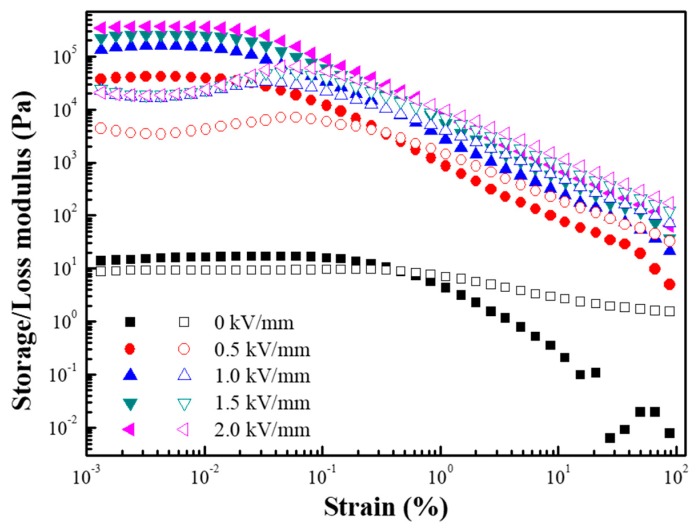
Storage (*G*′, closed symbols) and loss (*G*″, open symbols) moduli as a function of strain for PDPA/Fe_3_O_4_-based ER fluid (10 vol%, 50 cSt silicone oil) with constant angular frequency of 6.28 rad/s.

Oscillation tests were also carried out in a controlled shear deformation mode. Initially, the strain amplitude sweep tests were measured with a constant angular frequency (6.28 rad/s) to search the linear viscoelastic (LVE) region [51]. Figure 6 presents the strain-dependent storage (*G*′) and loss (*G*″) moduli of the PDPA/Fe_3_O_4_ ER suspension. In the low strain range, *G*′ showed a plateau region, called the LVE region, where the chain-like forms were strong enough not to be destroyed by the applied strain. When the strain exceeded the critical value, structures that had formed began to be destroyed, showing a sharp decline in *G*′. Therefore, to avoid excessive strain causing chain damage and affecting the frequency-dependent changes in the chain structures, a strain value of 0.007% in the LVE region was selected as constant strain in the following frequency sweep tests.

**Figure 7:**
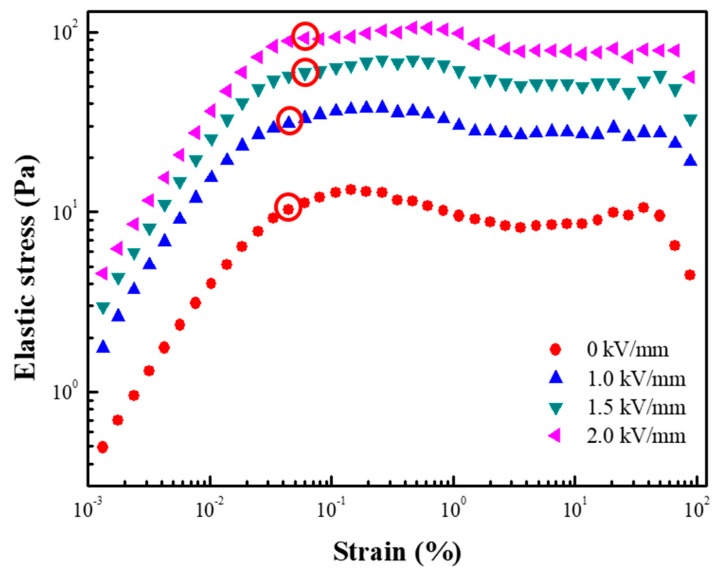
Elastic stress as a function of strain for PDPA/Fe_3_O_4_-based ER fluid (10 vol%, 50 cSt silicone oil). The red circles indicate the yield points.

The elastic stress ($\tau_{e}$) from the dynamic oscillation test can be calculated using Equation (5):

(5) $$\tau_{e}\;=\;G^{\prime }\cdot \gamma$$

where γ is the strain value. As given in Figure 7, at the low strain range, the elastic stress increased with increased shear strain, where the deformation of the chain structures could be restored after the stress was removed, indicating that the chain structures were elastic within this range. However, when the strain exceeded a critical value, the elastic stress showed a short plateau region and then decreased with increasing strain. The point at which the elastic stress showed the maximum value is defined as the yield point, and the elastic stress at this value is known to be the elastic yield stress [52,53]. The red circles in Figure 7 indicate the yield points.

**Figure 8:**
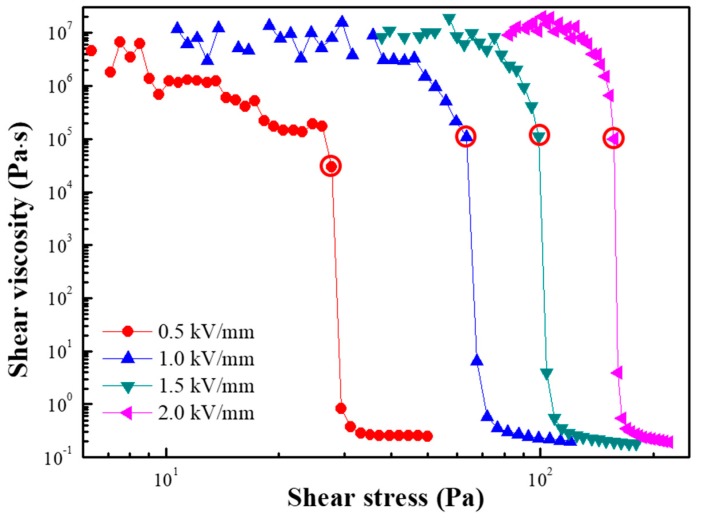
Shear stress-dependent shear viscosity of PDPA/Fe_3_O_4_-based ER fluid (10 vol%, 50 cSt silicone oil). The red circles label the static yield stress.

The static yield stress ($\tau_{sy}$) is defined as the minimum shear stress that causes the chain structures to begin to slide [54], which is different from the dynamic yield stress (i.e., the minimum stress that is required to continuously break the chain structures). Figure 8 demonstrates the dependence of shear viscosity on shear stress measured in a controlled shear stress (CSS) test mode where the shear stress exhibits a sudden decrease in shear viscosity when it reaches a certain value. The shear stress value at this point is regarded as $\tau_{sy}$.

**Figure 9:**
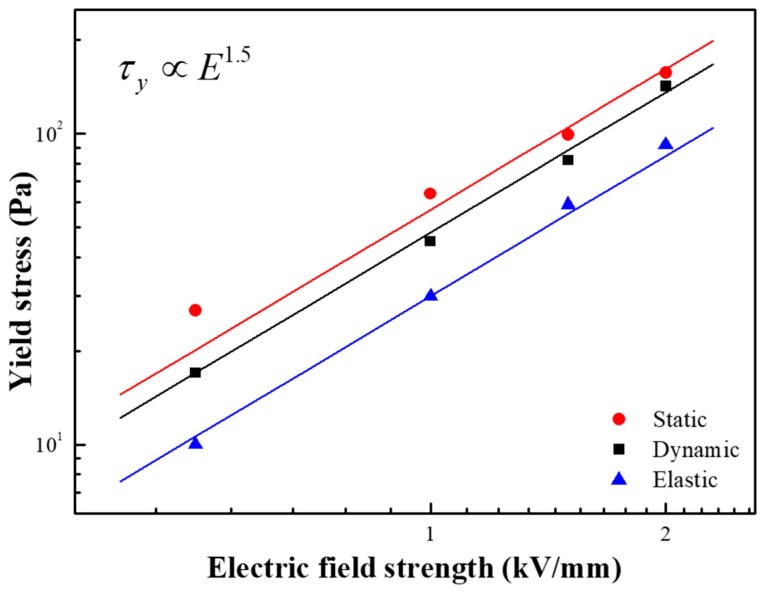
Elastic, dynamic, and static yield stress under various electric field strengths for PDPA/Fe_3_O_4_-based ER fluid (10 vol%, 50 cSt silicone oil).

Figure 9 presents the yield stress as a function of an input electric field, where Figure 7, Table 1, and Figure 8 show the elastic, dynamic, and static yield stresses, respectively. The correlation between the three types of yield stress and electrical field strength (*E*) generally follows a power-law expression:

(6) $$\tau_{y}\propto E_{m}$$

where the power law index, *m*, varies typically between 1.0 and 2.0, depending on the concentration of the ER fluids, morphology, size, dielectric, and conductivity of the different ER materials [15]. For the PDPA/Fe_3_O_4_ ER suspension in this work, the slope was observed to be *m* = 1.5 for all three different yield stresses, conforming to the conduction model proposed by Davis and Ginder [55], where the yield stress of an ER suspension is expressed proportional to the $E^{3/2}$.

**Figure 10:**
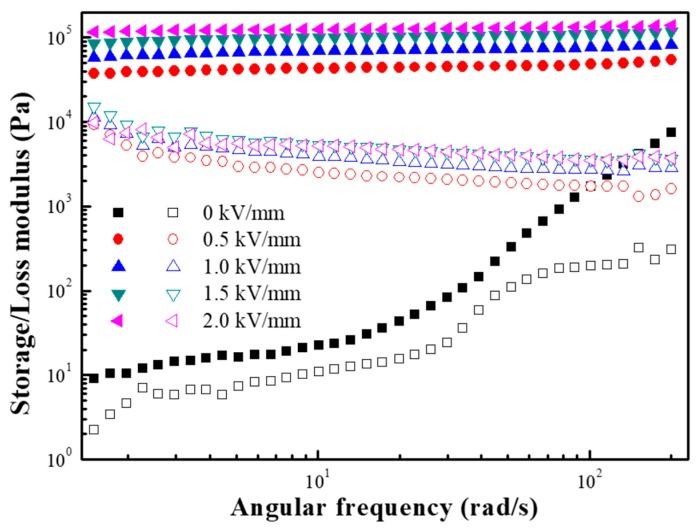
Storage (*G*′, closed symbols) and loss (*G*″, open symbols) moduli as a function of angular frequency for PDPA/Fe_3_O_4_-based ER fluid (10 vol%, 50 cSt silicone oil) with constant strain of 0.007%.

The frequency sweep measurement was tested with an angular frequency, varying from 0.1 and 200 rad/s at a constant strain (0.007%). As given in Figure 10, without an electrical field, both *G*′ and *G*″ increased with an increased angular frequency, but with an input electrical field, they exhibited a relatively stable value over a whole frequency region and increased with increasing electric field strength. This indicated that under an input electric field, the interactions between the particles became stronger and the chain structures were not easily broken. Furthermore, a time-dependent relaxation modulus *G*(*t*) can be estimated from the frequency sweep test using the following Schwarzl equation [56]:

(7) $$G\left(t\right)\;≅\;G^{\prime }\left(\omega \right)\;-\;0.566G^{\prime \prime }\left(0.5\omega \right)\;+\;0.203G^{\prime \prime }\left(\omega \right).$$

**Figure 11:**
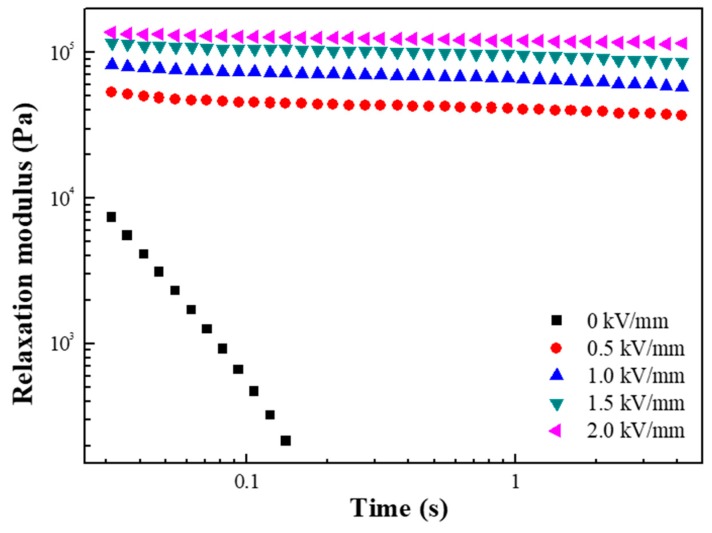
Time-dependent relaxation modulus of PDPA/Fe_3_O_4_-based ER fluid (10 vol%, 50 cSt silicone oil).

Figure 11 presents the time-dependent relaxation modulus under different electrical field strengths. In the absence of electric field, *G*(*t*) decreased significantly in a very short period of time, indicating that the ER suspension exhibited liquid-like properties. Meanwhile, with an electrical field, the relaxation modulus exhibited a stable value, showing that the ER fluid became a solid-like state.

In summary, it is not difficult to confirm that the PDPA/Fe_3_O_4_-based ER fluid changes from a fluid-like state to a solid-like state under the stimulation of an electric field based on the results of the static shear tests and the dynamic oscillation tests. Moreover, under the electric field, with the increase of the shear rate, the ER fluid exhibited a very stable shear stress curve, such that the yield stress was up to 100 Pa, and the ER efficiency was also very high. The relatively high and stable behaviors of PDPA/Fe_3_O_4_-based ER fluid may increase the possibility of its practical application in engineering.

#### 3.2.4. Dielectric Properties

In addition, the frequency-dependent dielectric behaviors of the ER suspension are important for predicting its ER effects. To further study the relationship among them, the dielectric constant ($\varepsilon^{\prime }$) and loss factor ($\varepsilon^{\prime \prime }$) of the PDPA/Fe_3_O_4_-based ER suspension over the wide frequency region (from 20 to 10^6^ Hz) were tested using a LCR meter. Note that dielectric relaxation of materials can be described simply using the following Cole–Cole equation [57,58]

(8) $$\varepsilon *\;=\;\varepsilon^{\prime }\;+\;i\varepsilon^{\prime \prime }\;=\;\varepsilon_{\infty }+\frac{\varepsilon_{0}-\varepsilon_{\infty }}{1+{\left(i\omega \lambda \right)}^{1-\alpha }};\;0\leq \alpha <1.$$

According to this equation, the dielectric constant ($\varepsilon^{\prime }$) and loss factor ($\varepsilon^{\prime \prime }$) can be expressed as

(9) $$\varepsilon^{\prime }\;=\;\varepsilon_{\infty }\;+\left(\varepsilon_{0}{-\varepsilon }_{\infty }\right)\frac{{1+(\omega \lambda )}^{1-\alpha }cos\frac{\pi (1-\alpha )}{2}}{{1+2(\omega \lambda )}^{1-\alpha }cos\frac{\pi (1-\alpha )}{2}{+(\omega \lambda )}^{2(1-\alpha )}}$$

(10) $$\varepsilon^{\prime \prime }\;=\;\frac{{(\varepsilon }_{0}{-\varepsilon }_{\infty }){\left(\omega \lambda \right)}^{1-\alpha }sin\frac{\pi (1-\alpha )}{2}}{{1+2(\omega \lambda )}^{1-\alpha }sin\frac{\alpha \pi }{2}{+(\omega \lambda )}^{2(1-\alpha )}}$$

where ε* is the complex dielectric constant; $\varepsilon_{0}$ and $\varepsilon_{\infty }$ are the dielectric constants as the frequency approaches to zero and infinity, respectively; ω = 2πf, f is the frequency; λ represents the polarization relaxation time; the exponent α takes a value between 0 and 1, which can describe different polarization relaxation situations.

**Figure 12:**
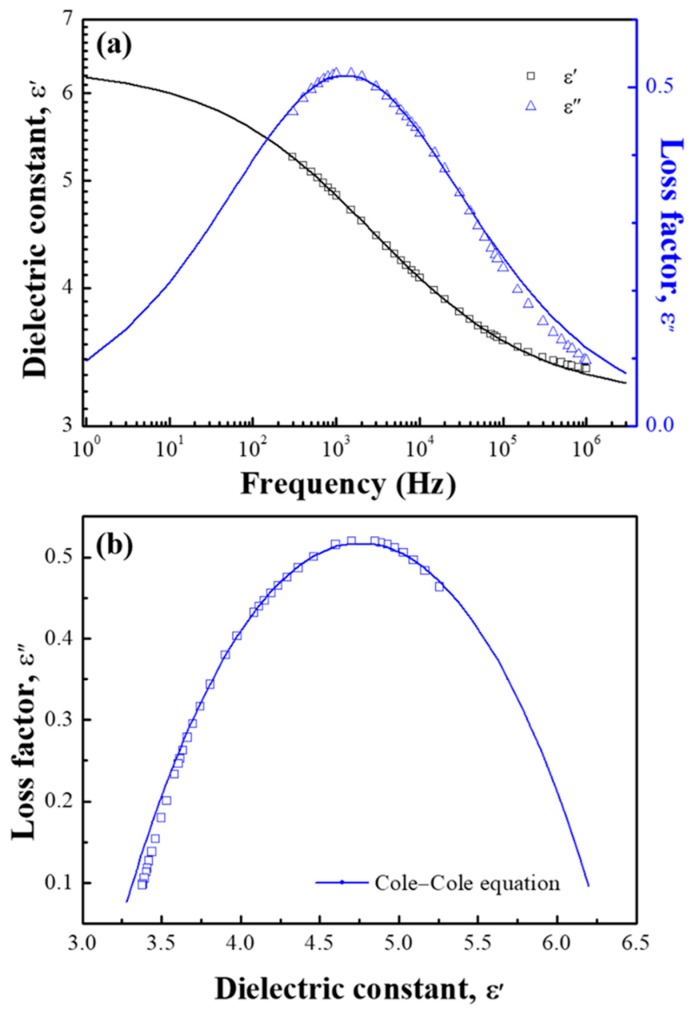
Dielectric properties: (**a**) dielectric constant (square) and loss factor (triangular) vs. frequency and (**b**) loss factor vs. dielectric constant for the PDPA/Fe_3_O_4_-based ER fluid (10 vol%, 50 cSt silicone oil). Solid lines were drawn from Cole−Cole equation.

Figure 12 gives the dielectric values of the PDPA/Fe_3_O_4_ ER fluid, in which the solid lines were fitted using Cole–Cole equation with their fitted parameters shown in Table 2. The value of ∆ε is related to the electrostatic interactive forces among the dispersed particles, which can be seen as the achievable polarizability of an ER suspension. The polarization relaxation time (λ) reflects the ER effect, in which the faster relaxation time generally contributes to the higher yield stress enhancement [59]. The exponent (1−α) is associated with the distribution broadness of the polarization relaxation time, and when α = 0, it indicates a single relaxation time. As shown in Table 2, the ∆ε value of PDPA/Fe_3_O_4_-based ER fluid was 3.07 and the relaxation time was fast enough to be 1.23 × 10^−5^ s, which indicated that the PDPA/Fe_3_O_4_ ER suspension had high polarizability and high yield stresses enhancement, which requires further study. Moreover, the ER tests showed that the conclusions inferred by dielectric properties were consistent with the results of the ER experiments. Meanwhile, compared with the PDPA-based ER fluid (∆ε = 0.62) [43], the ∆ε value of the PDPA/Fe_3_O_4_ ER suspension was significantly higher, which was consistent with the result of PDPA/Fe_3_O_4_-based ER fluid exhibiting higher shear stress, shear viscosity, dynamic moduli, and ER efficiency than the PDPA-based ER suspension with an electrical field.

In addition, magnetorheological (MR) fluids have similar properties to ER suspensions, which are usually composed of soft-magnetic particles dispersed in a carrier liquid, and its viscoelastic properties change significantly with the employment and withdrawal of the magnetic field. Because the as-fabricated PDPA/Fe_3_O_4_ is a soft magnetic material, it has also been studied as an MR material. The detailed contents can be found in previous work [60], where the PDPA/Fe_3_O_4_-based MR fluid provided a largely increased shear stress, shear viscosity, and dynamic moduli with the application of a magnetic field.

## 4. Conclusions

Electro-responsive composite (PDPA/Fe_3_O_4_) particles were fabricated by synthesizing PDPA using an oxidation polymerization method and loading nano-sized Fe_3_O_4_ particles onto it using chemical co-precipitation. The morphology and crystal structure of the fabricated PDPA/Fe_3_O_4_ was studied by SEM, TEM, and XRD. The formation of chain-like structures of PDPA/Fe_3_O_4_ particles with an external electrical field was characterized by optical microscopy. The rheological characteristics of the PDPA/Fe_3_O_4_ ER suspension under various electrical field strengths were investigated by a rotation rheometer. In the static shear tests, the PDPA/Fe_3_O_4_–based ER fluid demonstrated a Newtonian fluid property, while under an electrical field, the ER suspension demonstrated Bingham plastic behavior and was fitted well by the Bingham model. In the dynamic oscillation tests, with an input electrical field, the dynamic moduli showed a dramatic increase and provided proof of the transition of the ER suspension from a liquid-like to solid-like state. The relationship between yield stresses and electrical field strength showed that it conforms to the conduction model. The dielectric characteristics of the PDPA/Fe_3_O_4_ ER suspension also were tested via a LCR meter, corresponding to the ER characteristics. Furthermore, along with its MR characteristics previously reported, the PDPA/Fe_3_O_4_–based ER fluid can contribute to more stable ER performance than a PDPA–based ER fluid. Compared with the other previously reported organic–inorganic ER materials, the most significant advantage of the PDPA/Fe_3_O_4_ was that it had suitable conductivity, so that no de-doping step was needed, which undoubtedly improved the economic efficiency while simplifying the operation. All in all, the synthesis process of the PDPA itself was simple, the flow behavior of the PDPA/Fe_3_O_4_ ER fluid was stable, the ER efficiency was high, and the economic benefit was good; these advantages of the PDPA/Fe_3_O_4_ ER fluid are beneficial to its application in engineering.

## Figures and Tables

**Table 1:** Fitted parameters of Bingham model for PDPA/Fe_3_O_4_-based ER fluid (10 vol%, 50 cSt silicone oil).

Electric Field (kV/mm)	$\tau_{dy}$	$\eta_{\infty }$
0.5	16.6	0.11
1.0	40.2	0.10
1.5	79.9	0.09
2.0	133.7	0.08

**Table 2:** Fitted parameters of Cole−Cole equation for PDPA/Fe_3_O_4_-based ER fluid (10 vol%, 50 cSt silicone oil).

Parameters	$\varepsilon_{0}$	$\varepsilon_{\infty }$	$∆{\varepsilon \;=\;\varepsilon }_{0}\;{-\;\varepsilon }_{\infty }$	*λ* (s)	*α*
Value	6.34	3.17	3.07	1.23 × 10^−5^	0.598
